# Predicting Avian Influenza Co-Infection with H5N1 and H9N2 in Northern Egypt

**DOI:** 10.3390/ijerph13090886

**Published:** 2016-09-06

**Authors:** Sean G. Young, Margaret Carrel, George P. Malanson, Mohamed A. Ali, Ghazi Kayali

**Affiliations:** 1 Department of Geographical and Sustainability Sciences, University of Iowa, Iowa City, IA 52242, USA; margaret-carrel@uiowa.edu (M.C.); george-malanson@uiowa.edu (G.P.M.); 2 Department of Epidemiology, University of Iowa, Iowa City, IA 52242, USA; 3 Center of Scientific Excellence for Influenza Viruses, National Research Centre, Giza 12311, Egypt; mohamedahmedali2004@yahoo.com; 4 Department of Epidemiology, Human Genetics, and Environmental Sciences, University of Texas Health Sciences Center, Houston, TX 77030, USA; ghazi@human-link.org; 5 Department of Scientific Research, Human Link, Hazmieh 1107-2090, Lebanon

**Keywords:** coinfection, Egypt, influenza, remote sensing, ecological niche modeling, geography, medical, influenza A virus, H5N1 subtype, influenza A virus, H9N2 subtype

## Abstract

Human outbreaks with avian influenza have been, so far, constrained by poor viral adaptation to non-avian hosts. This could be overcome via co-infection, whereby two strains share genetic material, allowing new hybrid strains to emerge. Identifying areas where co-infection is most likely can help target spaces for increased surveillance. Ecological niche modeling using remotely-sensed data can be used for this purpose. H5N1 and H9N2 influenza subtypes are endemic in Egyptian poultry. From 2006 to 2015, over 20,000 poultry and wild birds were tested at farms and live bird markets. Using ecological niche modeling we identified environmental, behavioral, and population characteristics of H5N1 and H9N2 niches within Egypt. Niches differed markedly by subtype. The subtype niches were combined to model co-infection potential with known occurrences used for validation. The distance to live bird markets was a strong predictor of co-infection. Using only single-subtype influenza outbreaks and publicly available ecological data, we identified areas of co-infection potential with high accuracy (area under the receiver operating characteristic (ROC) curve (AUC) 0.991).

## 1. Introduction

Human infections with highly pathogenic avian influenza (HPAI) H5N1 and other subtypes are a continuing concern for global public health. Previous human outbreaks have been largely constrained by poor viral adaptation to non-avian hosts [1]. This limitation could, potentially, be overcome via reassortment, whereby two different strains co-infecting the same host share genetic material to create new hybrid strains [2]. Most previous pandemics in humans were the results of such reassortment events. Monitoring and mapping the movement of influenza viruses within bird populations and identifying areas of co-infection potential can help in targeting surveillance efforts and intervention campaigns [3]. Identification of ecological niches that are conducive to co-infection with different influenza subtypes can indicate areas where novel reassortants may emerge. We hypothesize there are such areas of overlapping niches and susceptible hosts in these places are at increased risk of co-infection.

HPAI H5N1 first emerged in Asia in 1996 and has since spread to become a panzootic. Due to its high pathogenicity (in both poultry and human hosts) and wide geographic spread, H5N1 is often considered one of the most likely pandemic candidates, with some insisting an H5N1 pandemic is inevitable [4]. However, not all previous pandemics arose from HPAI viruses. It has also been suggested H9N2, a low pathogenic avian influenza (LPAI) virus, which emerged in 1994, may be an equally plausible candidate for the next pandemic virus [1,5]. H9N2 viruses are endemic in poultry across a wide geographic range, have caused infections in humans and pigs, and are, at least in some cases, partially adapted to recognize and bind to human receptors [6,7]. It is conceivable that co-infection with H5N1 and H9N2 could produce a new subtype capable of infecting humans with high pathogenicity.

Strong evidence suggests aquatic birds act as the natural reservoir host for the influenza virus, and surveillance and intervention at the avian-human interface may be critical to the early identification and eradication of the next potentially pandemic strain [1,8]. Most human infections with avian influenza have been the result of direct contact with poultry, not wild birds, likely due to the much higher rate of contact [9]. The potential for direct contact between humans and poultry is high in Egypt. The majority of poultry production is carried out in commercial farms. Some 4–5 million Egyptian families raise poultry at home in traditional backyard flocks, accounting for roughly a third of poultry production in the country [10]. While commercial farms generally have high biosecurity limiting poultry contact with potentially infected wild birds, backyard farms are characterized by outdoor enclosures with little security. For example, a three-year study of backyard poultry growers in Egypt found that none of the participants (*n* = 750) consistently used or decontaminated personal protective equipment [11]. Egypt is an arid country in Northeastern Africa, containing just over one million square kilometers of land, of which less than 3% is arable, mostly located in the Nile River valley and delta [12]. Both human and poultry populations are most densely distributed in these arable regions [13].

HPAI H5N1 was identified in Egyptian poultry in early 2006, and resulted in the loss of more than 90% of flocks that year [14]. Since that time, Egypt has reported more human cases of H5N1 influenza than any other country, with 346 confirmed cases as of 31 December 2015 [15]. Egypt reported over 1000 outbreaks of H5N1 in poultry to the World Organization for Animal Health (OIE) before declaring the disease as endemic in July 2008, less than two years after its introduction [16]. The economic impacts have also been substantial. In addition to losses directly from sickness and death of infected birds, mandatory culling of entire flocks upon detection of the virus has been enforced without compensation for farmers, resulting in an estimated $2–3 billion USD in losses in the first four years [17].

Avian influenza subtype H9N2 was first identified in Egyptian poultry in 2011 and has caused confirmed human infections [18]. Active surveillance of Egyptian poultry has detected co-infection with both H5N1 and H9N2, which could potentially lead to reassortment [19]. As the spatial distribution of influenza in Egyptian poultry is not uniform, and considering the relative scarcity of co-infection data, we investigated a method to identify areas where poultry may be at increased risk of co-infection in the absence of true co-infection data.

Ecological niche modeling (ENM) and the related species distribution modeling (SDM) were developed by geographers and ecologists to predict the potential geographic occurrence of organisms based on observed occurrences in niche space [20]. We define the niche as the combination of environmental conditions (here, these include host populations and indirect indicators) within which an organism can persist. ENM and SDM are sometimes used interchangeably; however, modeling the processes that shape distributions and predicting the potential distribution in unsampled areas assumes a niche-based hypothesis, so we will use the term ENM (for a detailed discussion, see [21,22], but also see [23,24]). ENM is only possible on a broad scale with the use of remotely-sensed datasets, which tend to have much higher spatial and temporal resolution than observational data, and cover a considerably larger spatial extent [25,26]. Remotely-sensed data allow detailed modeling across very large regions, including areas where more traditional observational data are not available or are severely outdated, a common problem in many developing countries, such as Egypt.

In this paper we identify areas with increased potential of co-infection outbreaks in poultry flocks by modeling the ecological niches of H5N1 and H9N2 separately using known outbreak locations in Northern Egypt, then observing where these niches overlap in geographic space. This overlap is then compared with a niche model developed using actual co-infection occurrences to evaluate the models’ predictive ability. Additional models are developed to evaluate the transferability of niche estimates into unsampled locations.

## 2. Materials and Methods

**Figure 1:**
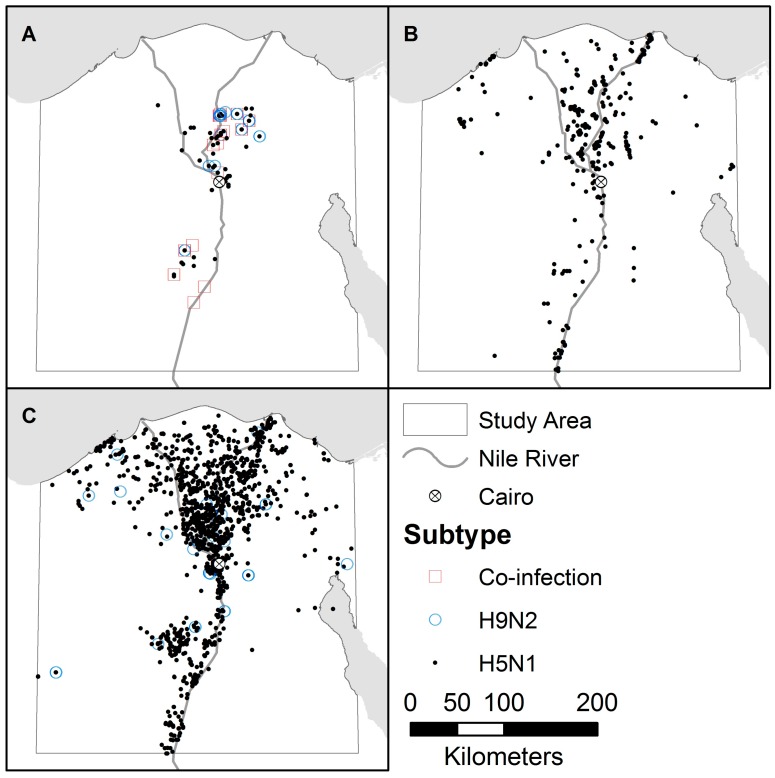
Distribution of influenza outbreaks by subtype from (**A**) the systematic surveillance program; (**B**) the World Organization for Animal Health (OIE); and (**C**) the Emergency Prevention System against transboundary animal and plant pests and diseases (EMPRES) Global Animal Disease Information System (EMPRES-i).

Three sources were used for outbreak data from 2007 to 2015: a systematic avian influenza surveillance program between the Center of Scientific Excellence for Influenza Viruses in Egypt and the St. Jude Center of Excellence for Influenza Research and Surveillance in the United States [27], the avian influenza database from the OIE [28] and the Emergency Prevention System against transboundary animal and plant pests and diseases (EMPRES) Global Animal Disease Information System (EMPRES-i) from the Food and Agriculture Organization (FAO) of the United Nations [29]. Data from the systematic surveillance program, ongoing since 2009, include monthly non-random samples for individual birds from commercial and backyard farms, as well as abattoirs and live bird markets (LBM). The OIE data, covering 2007 to February 2008, include reported outbreaks for flocks from commercial farms, backyard farms, and LBMs. The EMPRES-i data, from February 2008 to 2015, include reported outbreaks for flocks, but the majority do not record the habitat. The systematic surveillance program is the only data source that tests for and reports co-infection. For consistency, records for individual birds were aggregated into outbreaks based on location and date, with duplicates between data sources removed. Outbreaks were geocoded to the scale of individual farms, except for the systematic surveillance data which were geocoded to villages (see Figure 1). The bulk of the outbreaks from all three data sources were collected in seven governorates in Northern Egypt, representing the primary foci of the poultry industry [19].

The ecological dataset represents environment, population, and behavior variables relevant to the ecology of influenza, primarily collected via remote sensing. Several studies have successfully modeled avian influenza niches using environmental variables. Researchers modeling HPAI in Japan used temperature, precipitation, elevation, and poultry density, finding the last two most strongly correlated with the distribution of avian influenza [30]. Others modeled the ecological niche of avian influenza in the Middle East using only remotely-sensed vegetation indices and elevation data [31]. Models developed for Europe and India have also found slope, temperature ranges, and human population data to be useful [32,33]. Humidity has been shown to play a role in virus persistence and transmission effectiveness [34], and models of H5N1 in China, Vietnam, Thailand, and Madagascar used distance to fresh water and distance to major roads as predictive variables [35,36]. One study that developed a model for H5N1 in Egypt concluded that information on demographics related to poultry holdings is necessary to improve predictions [37].

Only publicly available datasets with wide geographic (preferably global) coverage and moderate spatial resolution were selected (see Table 1). Climatic and other remotely-sensed variables included relative humidity, precipitation, air temperature, surface temperature, elevation (and slope), and a vegetation index. Average annual relative humidity was obtained from the Center for Sustainability and the Global Environment (SAGE) at the University of Wisconsin-Madison’s Atlas of the Biosphere (AoB) database, and was selected for its impact on viral persistence and transmission effectiveness [34]. Total annual precipitation and monthly average air temperature came from the WorldClim database, both relevant to viral survival [30]. Mean seasonal and annual diurnal surface temperature and the Normalized Difference Vegetation Index (NDVI) were derived from remotely sensed data acquired by NASA’s Moderate Resolution Imaging Spectroradiometer (MODIS) sensor on the TERRA satellite [38]. Diurnal temperature may be associated with both viral persistence and with movements of migratory birds, a theorized source of viral diffusion [33,39]. NDVI is a measure of “greenness” or vegetation health, and represents host habitat suitability [31]. Data were downloaded in tiles and mosaicked together, then monthly values were aggregated to seasonal/annual values. Elevation and slope data were derived from NASA’s Shuttle Radar Topography Mission (SRTM) 30-meter digital elevation model (DEM) processed and distributed by the Consultative Group for International Agricultural Research (CGIAR) Consortium for Spatial Infrastructure (CGIAR-CSI), and influence habitat suitability and migratory pathways for waterfowl [30,33,35].

Distance to fresh water (important for waterfowl diffusion) and distance to major roads (important for poultry diffusion) were calculated, as well as distance to LBM within the study area, which may play a role in viral mixing and diffusion [33,35,41]. Both perennial water sources and roads data were obtained from the Defense Mapping Agency’s (DMA) Digital Chart of the World (DCW) available through the DIVA-GIS group [43]. These data were collected in 1992, so supplemental roads data derived from OpenStreetMap were also used [44]. The locations of LBM within the study area were derived from the systematic surveillance dataset, which yielded 76 markets and is believed to be representative.

Demographic information included populations of humans and poultry, a wealth index measurement, a measure of water scarcity, and bird husbandry variables including whether chickens and ducks were kept in separate flocks or allowed to mix, and if birds lived in or near homes. Human population data (2010, partially derived from remote sensing datasets) was obtained from WorldPop [45], and serves as an indication of the poultry trade [33,40]. Poultry density (2005) was obtained from the FAO’s Gridded Livestock of the World dataset, representing both commercial and backyard poultry [46]. Poultry density has been linked to viral transmission and diffusion [40]. The Egyptian Demographic and Health Survey (DHS) of 2008 provided the wealth index, information on the frequency of water scarcity, and bird husbandry behavior [13]. The wealth index and husbandry data are indications of biosecurity practices and the knowledge of and ability to implement appropriate disease mitigation strategies [32,42]. Water scarcity is important for habitat suitability, and may influence host diffusion and mixing [42]. Individual survey responses are geocoded to centroids of “DHS clusters” which represent spatial groupings of survey responses. Responses were aggregated to clusters and converted to a raster surface using empirical Bayesian kriging in ArcGIS 10.3 (Esri, Redlands, CA, USA) [47].

Most of the raster data was subset from global datasets at a resolution of 30 × 30 arcseconds, corresponding to about 0.8 square kilometer grid cells at the equator. Humidity and poultry density raster layers were of lower resolution (5 and 3 arcminutes respectively), but were resampled to match the spatial resolution of the other layers.

**Figure 2:**
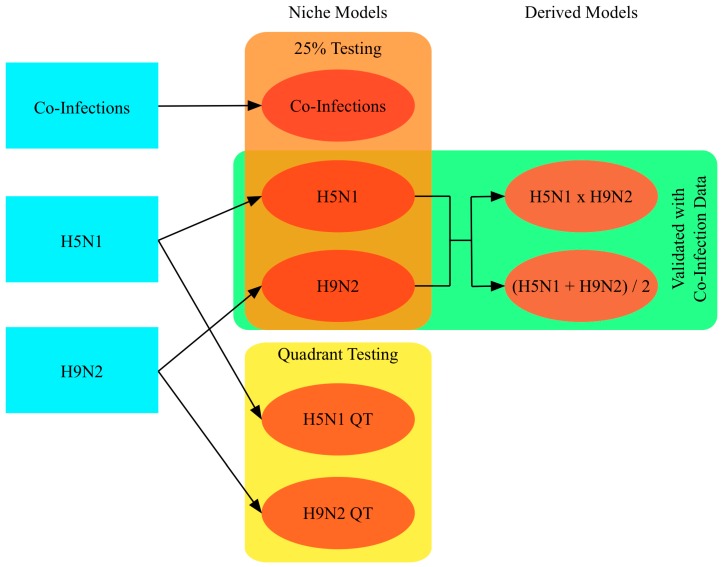
Conceptual diagram showing the creation of five niche models and two derived models, with rectangles indicating data used for model testing or validation.

We employed ENM to identify a set of environmental conditions associated with virus persistence and spread [48]. ENM examines the environmental data at locations of outbreaks and creates a model of the virus’ niche, which is evaluated and improved using randomly selected background points within the study area [49]. A large number of modeling algorithms are available with varying strengths and weaknesses, including regression-based models and machine learning methods [50,51]. We chose the maximum entropy modeling algorithm, which is robust to multicollinearity among input variables, and has been found to provide more conservative estimates of species niches than other common algorithms, including the Genetic Algorithm for Rule-set Production (GARP) and bioclimate analysis and prediction system (BIOCLIM), even with small sample sizes [52,53,54,55]. Presence-only models were created for outbreaks between 2007 and 2015 in Northern Egypt according to virus subtype, and co-infection potential maps were derived from them (see Figure 2).

Two main subtype models were created, one for H5N1 outbreaks, and one for H9N2 outbreaks, with known co-infection outbreaks omitted from preliminary analyses for subsequent evaluation of model performance. Models were parameterized to use 75% of outbreak locations for training and withhold 25% for testing, with 10,000 randomly selected background points as pseudo-absence locations. Niche models are sensitive to the locations of training points and have a tendency to over-fit the data. To evaluate the transferability of the niche estimates (i.e., how useful the estimates are outside the original study area) the study area was divided into quadrants with the first and third quadrants used for training and the second and fourth for testing, resulting in two additional models, one for H5N1 and one for H9N2 [54,56]. The niche estimates for the two subtypes, H5N1 and H9N2, were also combined to identify areas where hosts may be more susceptible to co-infection [57]. Two combinations were performed, multiplication and averaging. Multiplication creates a more focused map, and has the benefit of preserving “true zeros” where we would not expect to find one of the subtypes, making co-infection very unlikely. Averaging allows for high values in one niche to compensate for low values in the other, resulting in a larger area being assigned high co-infection potential. An independent dataset of actual co-infection occurrences during the study period was also used to create a co-infection niche model for comparison with the derived maps. A total of seven models were created: two for H5N1, two for H9N2, one for co-infection, and two models derived by combining the H5N1 and H9N2 models. Additional models that could not be properly validated were also created and are reported in the Supplemental Materials.

For all models a maximum of 10,000 iterations were performed to allow time for convergence. Jackknife tests were also performed where each variable was removed from the model in turn, and also used alone, to evaluate each variable’s importance to the model. Models were evaluated using the receiver operating characteristic (ROC) curve and the corresponding area under the curve (AUC) values. The ROC curve plots the true positive rate (sensitivity) against the false positive rate (1-specificity) at various thresholds, and the AUC measures the overall strength of the model, with values below 0.5 regarded as worse than a random model and values close to 1 representing a near-perfect model. One hundred bootstrap AUC calculations were performed to estimate the standard error and bias of these measures.

Niche equivalency and background similarity tests were performed for pairs of niche estimates using the I metric proposed by Warren et al. (2008), based on Hellinger distances, carried out using the phyloclim (v 0.9-4) package in R [58,59]. I ranges from 0 (no overlap) to 1 (niches are identical). *p*-values and confidence intervals (CI) for I were based on 100 permutations, shown previously to be sufficient to reject the null hypothesis of niche equivalence with high confidence [59]. Statistically significant results indicate the niche models tested are not identical/equivalent. The CI show the range of I values expected by chance, so values falling outside the CI indicate the niche models are more similar (above the CI) or different (below the CI) than expected by chance.

## 3. Results

By host, the majority of outbreaks were reported among chickens (*n* = 974), with the remainder made up of ducks (*n* = 395), mixed chickens and ducks (*n* = 725), other species, including geese, pigeons, quails, and turkeys (*n* = 77), or unspecified (*n* = 421). By habitat, outbreaks were recorded at backyard farms (*n* = 275), commercial farms (*n* = 202), live bird markets (*n* = 45), or other/unspecified (*n* = 2070) (see Table 2). The large number of outbreaks for which habitats and/or hosts were not recorded, exacerbating already small sample sizes when divided by subtype, made validating results of these models unreliable, however they are reported in the Supplemental Materials.

The AUC results for all of the models are reported in Table 3. The maps of co-infection potential created by multiplying and averaging the H5N1 and H9N2 niche estimates were evaluated using actual co-infection occurrences that were independent from the datasets used during training. The co-infection occurrences (*n* = 57) were used as presence locations vs. randomly selected background points in AUC calculations for the H5N1 niche, the H9N2 niche and the combined co-infection potential maps. The H5N1 niche had an AUC of 0.9276, the H9N2 niche had an AUC of 0.9379, and the H5_H9 niche had an AUC of 0.9918. Testing against co-infection occurrences, the H5N1 niche had an AUC of 0.9784 while the H9N2 niche had an AUC of 0.9923. The co-infection potential map using multiplication achieved an AUC of 0.9948, while the map using averaging of H5N1 and H9N2 achieved an AUC of 0.9939.

**Figure 3:**
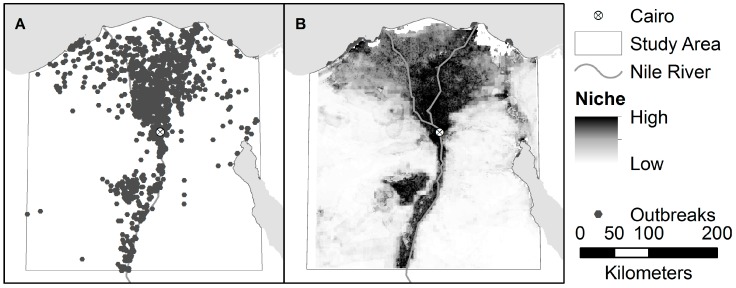
(**A**) Locations of H5N1 Outbreaks in the study area; and (**B**) the accompanying niche model for H5N1.

**Figure 4:**
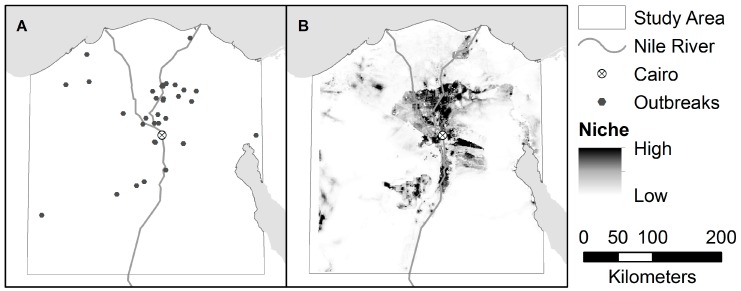
(**A**) Locations of H9N2 Outbreaks in the study area; and (**B**) the accompanying niche model for H9N2.

**Figure 5:**
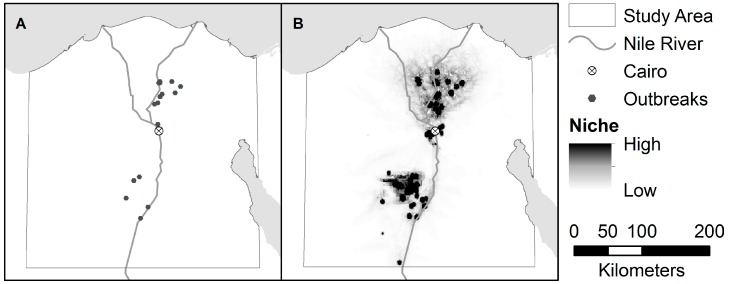
(**A**) Locations of co-infections of H5N1 and H9N2 in the study area; and (**B**) the accompanying niche model for co-infection cases.

**Figure 6:**
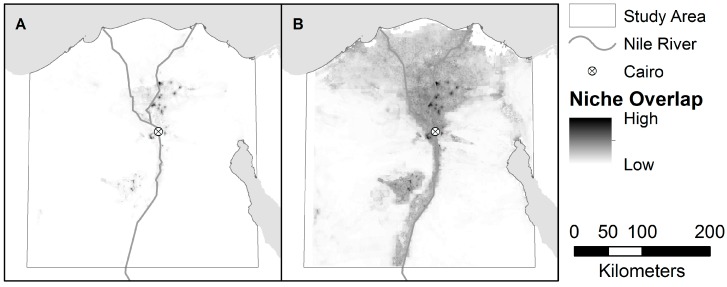
Maps of co-infection potential derived (**A**) by multiplying the H5N1 and H9N2 niche estimates; and (**B**) by averaging the H5N1 and H9N2 niche estimates.

The estimated distribution of areas suitable for H5N1 and H9N2, as predicted by the niche models, are shown in Figure 3 and Figure 4. The niche estimate for co-infection cases is depicted in Figure 5. Figure 6 shows the maps of co-infection potential derived from the niche estimates for H5N1 and H9N2 by multiplication and averaging. Niche equivalency and background similarity test results are shown in Table 4. All model comparisons using the I metric (H5N1 vs. H9N2, H5N1 vs. co-infection, and H9N2 vs. co-infection) were less than 1 and statistically significant and, therefore, non-equivalent.

**Figure 7:**
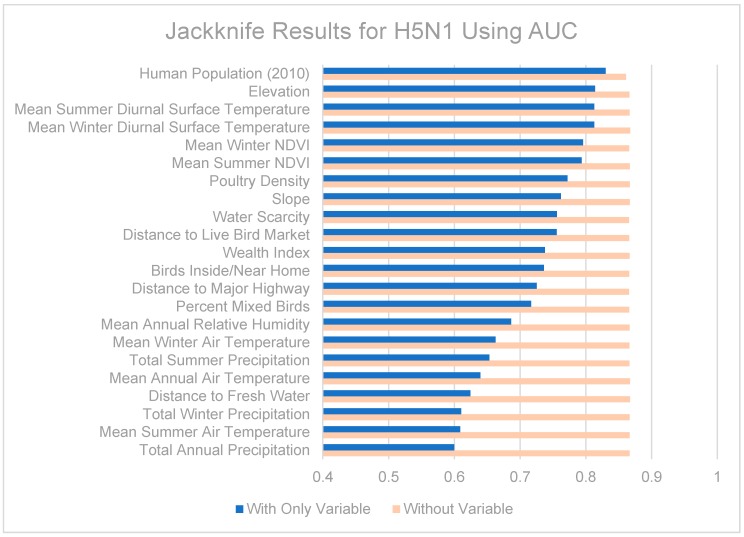
Jackknife test results using area under the receiver operating characteristic (ROC) curve (AUC) for H5N1.

**Figure 8:**
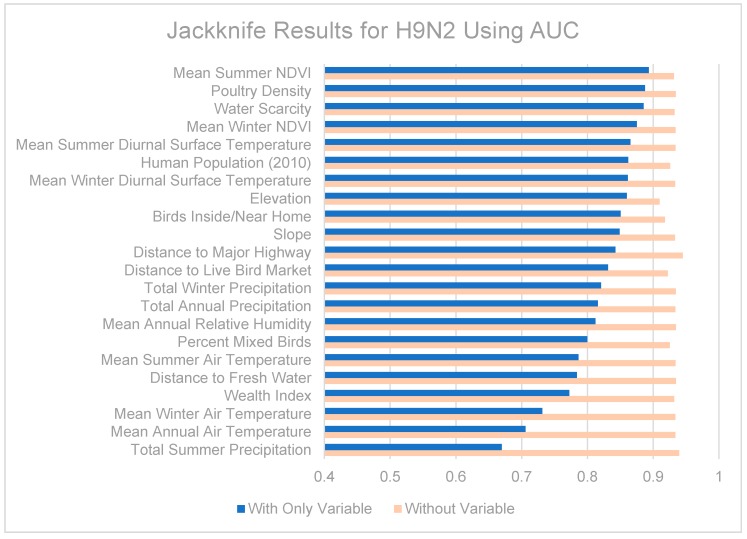
Jackknife test results using AUC for H9N2.

**Figure 9:**
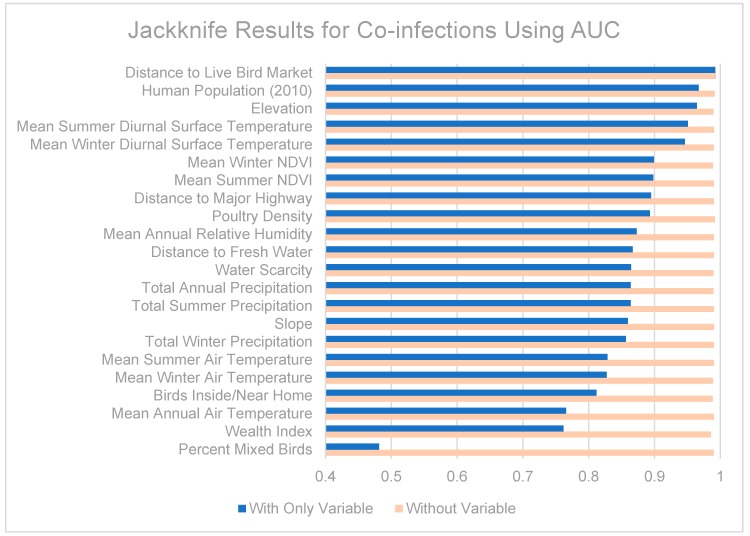
Jackknife test results using AUC for co-infections.

Jackknife tests are summarized in Figure 7, Figure 8 and Figure 9. The strongest single contributor to the H5N1 niche was human population, which was also the variable that caused the largest loss in AUC when it was removed from the model. Mean diurnal surface temperature (summer and winter), Elevation, and mean NDVI (summer and winter) were also strong contributors. For the H9N2 model, the strongest single contributor was mean summer NDVI, followed by Poultry density and water scarcity. The largest drop in AUC accompanied the removal of elevation from the model. The highest overall AUC for the H9N2 model was achieved by the full model after removing Distance to major highways. In both models, air temperatures, precipitation measurements, and distance to fresh water were generally poor contributors. The strongest contributor to the co-infection niche was distance to LBM, followed by human population and elevation. The largest drop in AUC coincided with the removal of the wealth index.

## 4. Discussion

The AUC for the co-infection potential maps were higher than the corresponding co-infection-AUC for individual subtype niche models, demonstrating that the method here outlined of combining single-subtype niche estimates is more effective at predicting co-infection occurrences than using single-subtype estimates alone. Furthermore, the derived maps were marginally more effective than the niche estimate created using co-infection cases, with a higher predicted AUC and smaller standard error.

All models achieved better than random AUC scores (i.e., above 0.5). Transferability was evaluated via the quadrant analyses. H9N2 resulted in an AUC drop from 0.9379 for the full model to 0.6902 by quadrants, indicating some overfitting of the training data. Similarly, the quadrant analysis for H5N1 resulted in an AUC drop from 0.9276 to 0.7413. These differences were significant (*t*-test, both *p* < 0.001), yet the AUC values for the quadrant models were still above 0.5, suggesting the models could be used in other study areas with environments similar to Northern Egypt, such as Northern Nigeria.

As expected, the map derived using multiplication resulted in a much narrower area of co-infection potential when compared with the averaged map (see Figure 5). Multiplying the single subtype niche estimates produces a more conservative map, only highlighting areas that were considered highly suitable in both models. This may more accurately reflect co-infection potential since we would not expect co-infection to occur in an area that’s only likely to support one subtype. The averaging method produces a more encompassing map, recognizing co-infections may occur outside the overlapping areas of the identified niches. In this study both maps were effective at predicting actual co-infection cases, but the multiplication-based map achieved a higher AUC with a lower standard error. The averaged map seemed to over-estimate the area of co-infection potential, resulting in its lower AUC and higher standard error, but there are times when this behavior might be desirable, (e.g., planning containment strategies). For intervention planning purposes, both maps are likely to be useful.

The niche equivalency tests showed the H5N1, H9N2, and co-infection niche models were not identical, but the background similarity tests demonstrated non-reciprocal relationships. The H5N1 and H9N2 models were more similar than expected by chance, the co-infection model was more similar to H5N1 and H9N2 than expected by chance, but the H5N1 and H9N2 models were more different from the co-infection model than expected by chance. Since the test compares probability distributions for one model against a randomized background derived from the second model, this result may simply reflect the nature of the co-infection niche as a “subset” of the single subtype niches. The smaller sample size of the co-infection niche may also play a role. In general, we would expect the different subtype niches to be more similar than expected by chance.

The contributions of the different variables to the niche models are somewhat difficult to tease out. When predictor variables are correlated (as some of those included are), results from jackknife tests should be interpreted with caution. Among the various models, remotely-sensed datasets made up the majority of the strongest contributing variables: human population (partially derived from remote sensing data), mean seasonal diurnal surface temperatures, elevation, and mean seasonal NDVI were all consistently strong contributors to the models. It may also be helpful to know which variables were poor contributors and can be omitted from future modeling efforts, including distance to fresh water and air temperature. Precipitation was also a moderate to poor predictor in our models, in contrast to recent modeling efforts in the Middle East which found seasonal precipitation to be the most important predictor for H5N1 [37]. This could be a result of the coarse spatial resolution used in the previous study (9 km^2^ compared to ~0.8 km^2^), or perhaps reflect our inclusion of NDVI which better captures vegetation health and available moisture than precipitation.

The H5N1 and H9N2 models differed markedly, as can be seen from their niche estimates (see Figure 3 and Figure 4) and jackknife results (Figure 7 and Figure 8). The H5N1 niche closely follows the Nile River and delta, with the desert oasis of Fayoum (southwest of Cairo) and portions of the Mediterranean coast having high H5N1 outbreak potential. The H9N2 niche, in contrast, appears more irregular and covers a much smaller geographic footprint. Human population is the strongest predictor for H5N1, but ranks sixth for H9N2, while mean summer NDVI is the most important predictor for H9N2, but ranks sixth for H5N1. Among their top five predictors, H5N1 and H9N2 only share two in common, mean summer diurnal surface temperature and mean winter NDVI. It is not yet clear what is causing these differences, although niche estimates differing by subtype has been reported previously [57]. It is possible that some of the differences are attributable to the fact H9N2 (2011) emerged more recently than H5N1 (2006) and may not yet be in equilibrium with the environment; that is, H9N2 may still be spreading geographically compared to the more saturated and stable H5N1 distribution, which would increase the uncertainty associated with the model [23,50]. A larger dataset covering a longer time period is required to properly address this possibility and assess the spatial and environmental stability of the niches over time.

Co-infection potential appears to be highest in areas with low to medium human population, moderate mean seasonal diurnal surface temperatures, low elevations, and low mean seasonal NDVI values. Distance to LBM was only a moderate contributor to the single-subtype models, but was the single best predictor for the co-infection model. This may indicate LBMs play a role in facilitating co-infection, a possibility supported by previous studies in Thailand and China, and one which deserves further investigation [60,61].

Sampling biases in the influenza surveillance datasets are a continuing problem. Outbreaks from backyard farms are underreported, and the non-random nature of the systematic surveillance sampling likely impacts the results of the current work. In practice, perfect surveillance programs are unrealistic and researchers are required to make do with available data. Results from this work may help demonstrate shortcomings of current surveillance efforts in Egypt, particularly by presenting areas where outbreaks are predicted yet sampling is sparse or non-existent.

It should be remembered that several of the models were constrained by small sample sizes, and therefore are less generalizable in their results. The standard error (SE) estimates accompanying the AUC results are an indication of the reliability of the models, with the H5N1 model having the smallest SE, and the H9N2 model having the largest. The limited precision of the geographic coordinates recorded for the co-infection outbreaks, and the resolution of the environmental data restricted analysis to approximately the village scale. Furthermore, while influenza infections spread very rapidly within flocks, co-infection within poultry flocks does not guarantee co-infection within individual birds, a prerequisite for reassortment events [62]. Despite these limitations, the methods outlined were able to predict locations with increased co-infection potential with a high degree of accuracy, suggesting similar efforts directed towards future outbreaks may be able to do the same.

## 5. Conclusions

This work contributes to our understanding of the role of landscapes on the ecology of the avian influenza virus in Egypt. Using primarily remotely-sensed data and ecological niche modeling we identified environmental, behavioral, and population characteristics which distinguish the niches of H5N1 and H9N2 subtypes within Egypt. H5N1 and H9N2 were found to differ markedly in their ecological niches and geographic distributions. Distance to LBM was found to be a strong predictor for the co-infection niche model. Using only single-subtype influenza outbreaks and publicly available ecological data, we identified areas of co-infection potential with high accuracy. Our method will enable researchers to quickly and inexpensively identify areas conducive to influenza co-infections, helping to target spaces for increased surveillance for novel strains and focusing limited resources during intervention campaigns.

The importance of accurately targeted influenza surveillance efforts and associated early-intervention strategies cannot be overstated [27]. The methods outlined provide valuable tools for finding useful environmental predictors and identifying areas where co-infection potential is highest. The theory and techniques used can be applied to avian influenza in other localities, as well as other infectious diseases with similar social and environmental drivers.

## Figures and Tables

**Table 1:** Environmental/ecological data resolutions and sources.

Dataset	Potential Relevance	Spatial Resolution	Timeframe	Source
Relative Humidity	Viral Persistence and Transmission [34]	5 arcminutes	1960–1990	Center for Sustainability and the Global Environment (SAGE) Atlas of the Biosphere (AoB)
Mean Air Temp	Viral Persistence [30]	30 arcseconds	1960–1990	WORLDCLIM
Total Precipitation	Viral Persistence [30]	30 arcseconds	1960–1990	WORLDCLIM
Mean Diurnal Surface Temp	Viral Persistence and Diffusion [33,39]	3 arcminutes	2009–2014	Moderate Resolution Imaging Spectroradiometer (MODIS)
Normalized Difference Vegetation Index (NDVI)	Habitat Suitability for Hosts [31]	30 arcseconds	2009–2014	MODIS
Elevation	Diffusion of Hosts [30,35]	30 arcseconds	2000	Shuttle Radar Topography Mission (SRTM)
Population (Human)	Indication of Poultry Trade [33,40]	3 arcseconds	2010	WorldPop
Live Bird Markets	Viral Mixing and Diffusion [41]	3 arcseconds	2009–2015	Systematic Surveillance
Poultry Density	Viral Transmission and Diffusion [40]	3 arcminutes	2005	Food and Agriculture Organization (FAO)
Fresh Water and Roads	Viral & Host Diffusion [33,35]	1:1,000,000, 1:50,000	1992, 2015	Defense Mapping Agency’s (DMA) Digital Chart of the World (DCW), OpenStreetMap (OSM)
Wealth Index	Biosecurity and Disease Mitigation [42]	Demographic and Health Survey (DHS) Clusters	2008	DHS
Water Scarcity	Habitat Suitability and Host Diffusion [42]	DHS Clusters	2008	DHS
Poultry Husbandry	Biosecurity [32]	DHS Clusters	2008	DHS

**Table 2:** Breakdown of avian influenza outbreaks (percentages by row).

		H5N1	H9N2	Co-Infection	Total
Source	Systematic Surveillance	145	39	57	241
	World Organization for Animal Health (OIE)	287	0	0	287
	EMPRES-i	1997	67	0	2064
Habitat	Commercial	133 (65.84)	28 (13.86)	41 (20.30)	202
	Backyard	261 (94.91)	6 (2.18)	8 (2.91)	275
	Live-bird Market	32 (71.11)	5 (11.11)	8 (17.78)	45
	Other/Unspecified	2003 (96.76)	67 (3.34)	0	2070
Host	Chicken	822 (84.39)	100 (10.27)	52 (5.34)	974
	Duck	390 (98.73)	1 (0.25)	4 (1.01)	395
	Mixed	724 (99.86)	0	1 (0.14)	725
	Other/Unspecified	493 (99.00)	5 (1.00)	0	498
Total	-	2429 (93.71)	106 (4.09)	57 (2.20)	2592

**Table 3:** Area under the receiver operating characteristic (ROC) curve (AUC) for avian influenza niche models.

Dataset (Subset)	AUC	Bias	Standard Error
H5N1	0.9276	−0.0005	0.0035
H5N1 (Quadrant Test)	0.7413	0.0003	0.0087
H9N2	0.9379	0.0003	0.0260
H9N2 (Quadrant Test)	0.6902	−0.0049	0.0485
Co-Infections	0.9918	0.00008	0.0078
H5N1 × H9N2 ^1^	0.9948	−0.00002	0.0015
Mean H5N1 & H9N2 ^1^	0.9939	0.0004	0.0020
H5N1 ^1^	0.9784	−0.0006	0.0044
H9N2 ^1^	0.9923	0.0001	0.0022

^1^ Tested using co-infection occurrences.

**Table 4:** Niche equivalency and background similarity tests for niche models.

Comparison (*X* vs. *Y*)	*I Metric*	*X* vs. Random *Y*	*Y* vs. Random *X*
H5N1 vs. H9N2	0.892 ^1^	(0.673, 0.763)	(0.754, 0.762)
Co-Infection vs. H5N1	0.478 ^1^	(0.261, 0.272)	(0.677, 0.780)
Co-Infection vs. H9N2	0.514 ^1^	(0.185, 0.272)	(0.682, 0.789)

^1^ Significance: *p* < 0.001.

## Supplementary Materials

The following are available online at http://www.mdpi.com/1660-4601/13/9/886/s1, Figure S1: Niche models by habitat. (A) Commercial poultry farms; (B) Backyard poultry farms; (C) Live bird markets, Figure S2: Niche models by host. (A) Chickens; (B) Ducks; (C) Mixed (chickens and ducks), Figure S3: Jackknife results for commercial habitats, Figure S4: Jackknife results for backyard habitats, Figure S5: Jackknife results for LBM habitats, Figure S6: Jackknife results for chicken hosts, Figure S7: Jackknife results for duck hosts, Figure S8: Jackknife results for mixed (chicken and duck) hosts, Table S1: Niche equivalency and background similarity tests for selected niche models, Table S2: Area under the ROC curve (AUC) for avian influenza niche models.
