# Supplementary material for: Predicting Avian Influenza Co-Infection with H5N1 and H9N2 in Northern Egypt
Source: Int J Environ Res Public Health. 2016 Sep 6;13(9):886. doi: 10.3390/ijerph13090886 (PMC5036719; doi:10.3390/ijerph13090886)
Supplement: Supplementary file 1 [file ijerph-13-00886-s001.pdf]

Supplementary Materials: Predicting Avian Influenza Co-Infection with H5N1 and H9N2 in Northern Egypt

Sean G. Young, Margaret Carrel, George P. Malanson, Mohamed A. Ali and Ghazi Kayali

The H5N1 dataset was subdivided and analyzed with models developed for specific habitats and hosts. Models were created for three habitats (backyard, commercial, and live bird markets (LBMs), see Figure S1) and for three hosts (chickens, ducks, and mixed (chicken and duck in the same flock), see Figure S2). The H9N2 and co-infection datasets could not be so subdivided without compromising the statistical validity of model results due to small sample sizes. Especially troublesome was the EMPRES-i data, which frequently failed to record habitat information, requiring large amounts of data to be excluded from these analyses. The models were parameterized according to the methods outlined in the main text. The permutation importance test results were normalized to percentages, and jackknife results were evaluated using area under the receiver operating characteristic curve (AUC) values and grouped into weak (<0.7), moderate (0.7–0.8) and strong (>0.8) contributor categories. There were not enough co-infection outbreaks in the various categories to validate any of the models with co-infection data.

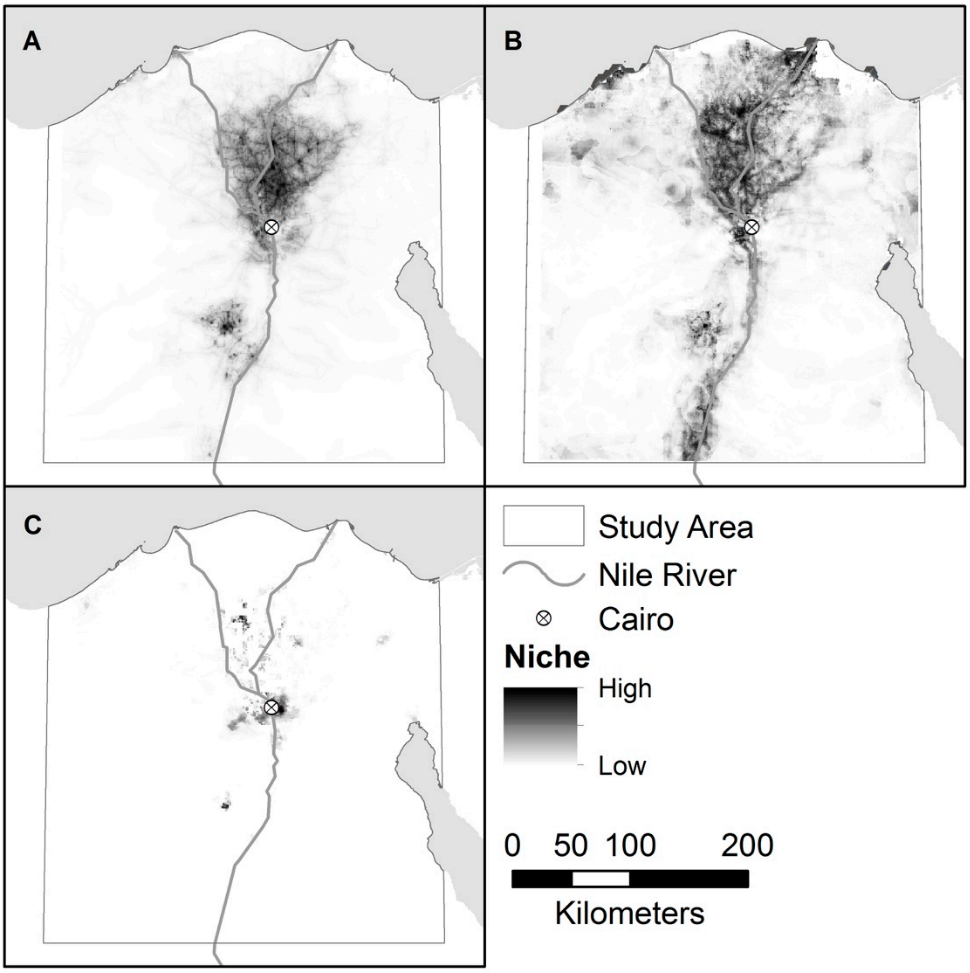

Figure S1. Niche models by habitat. (**A**) Commercial poultry farms; (**B**) Backyard poultry farms; (**C**) Live bird markets.

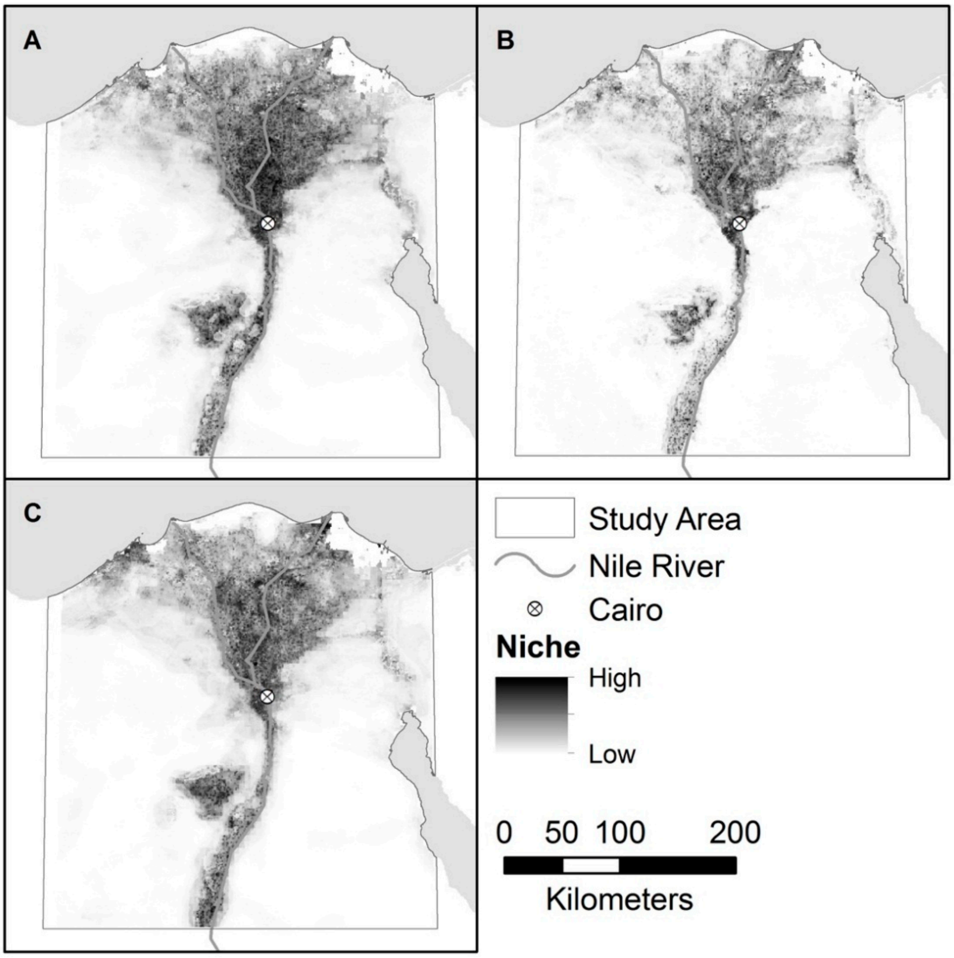

Figure S2. Niche models by host. (**A**) Chickens; (**B**) Ducks; (**C**) Mixed (chickens and ducks).

The equivalency and background similarity test results are shown in Table S1. The niche models created for backyard/commercial were not identical, but were more similar than would be expected by chance. Comparisons of backyard/LBM and commercial/LBM models were also not identical, but were more different than expected by chance. Finally, the chickens/ducks models were more similar than expected by chance, but the null hypothesis that the models were identical could not be rejected with the I metric. These results indicate that while the specific models compared were almost universally not equivalent; in most cases the niche models compared were more similar to each other than would be expected by chance. Subdividing the dataset into these categories therefore may not be worth the tradeoff of diminished sample sizes. Models for samples from LBM habitats may be an exception as they were more different from models for other habitats than expected by chance and may, therefore, provide insight into meaningful ecological differences that would be lost if the datasets were not so divided.

Table S1. Niche equivalency and background similarity tests for selected niche models.

Comparison (*X* vs. *Y*)	Result	*X* vs. Random *Y*	*Y* vs. Random *X*
H5N1: Backyard vs. Commercial	D: 0.557 **	(0.351, 0.450)	(0.289, 0.325)
	I: 0.823 **	(0.649, 0.757)	(0.587, 0.634)
H5N1: Backyard vs. LBM	D: 0.117 **	(0.289, 0.493)	(0.045, 0.062)
	I: 0.322 **	(0.563, 0.787)	(0.142, 0.185)
H5N1: Commercial vs. LBM	D: 0.167 **	(0.194, 0.346)	(0.031, 0.069)
	I: 0.434 **	(0.439, 0.654)	(0.099, 0.193)
H5N1: Chickens vs. Ducks	D: 0.812 *	(0.376, 0.424)	(0.330, 0.356)
	I: 0.964	(0.681, 0.726)	(0.629, 0.657)

Significance: * 0.05, ** 0.001.

According to the jackknife tests (see Figures S3–S8) mean summer and winter diurnal surface temperatures, elevation, and human population were strong contributors in every model. Mean summer and winter normalized difference vegetation index (NDVI) were also strong contributors for the majority of models. Mean summer air temperature, mean annual air temperature, total summer precipitation, and distance to fresh water were generally weak predictors. It is also interesting to note that human population alone was able to achieve a higher AUC score than the full model using all variables for the LBM niche. The similarity of the results between the models strengthens the argument that subdividing the outbreak dataset by habitat and host does not provide a wealth of insight, but rather results in a number of very similar models.

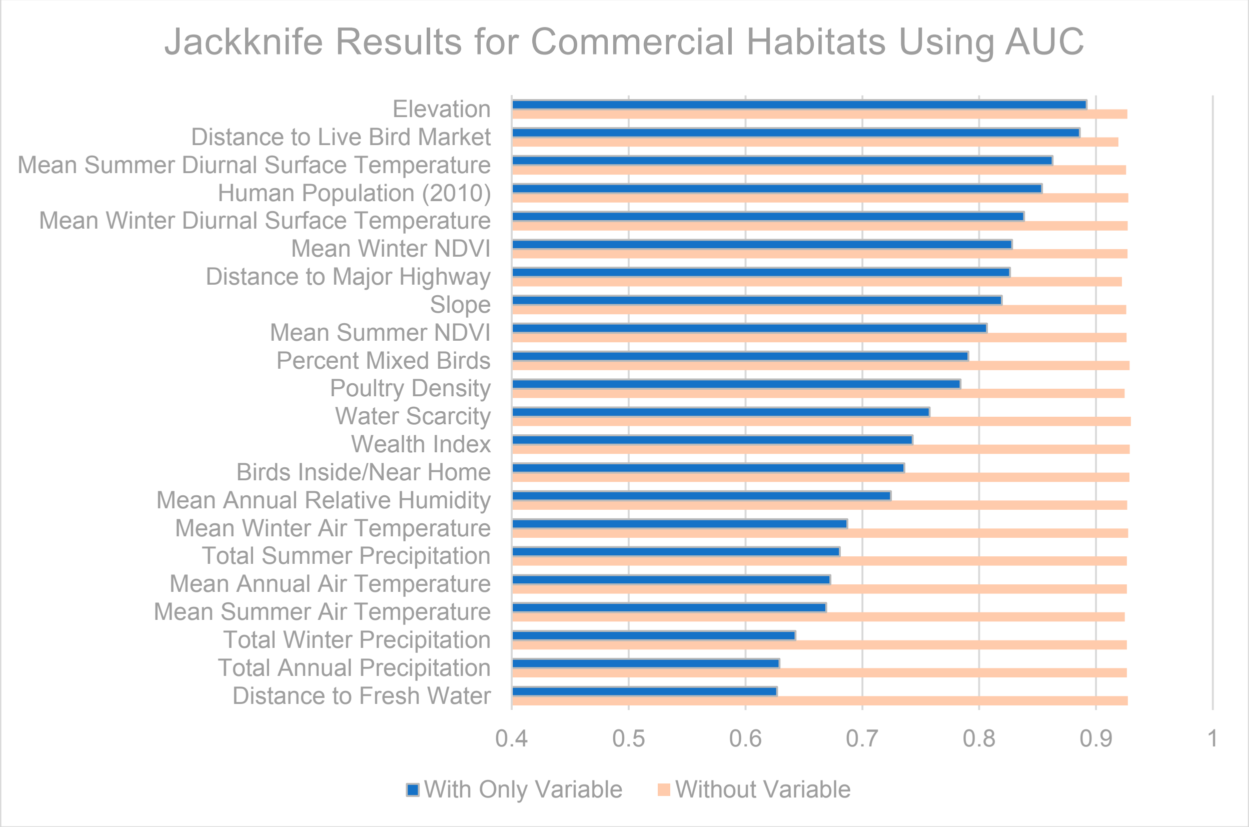

Figure S3. Jackknife results for commercial habitats.

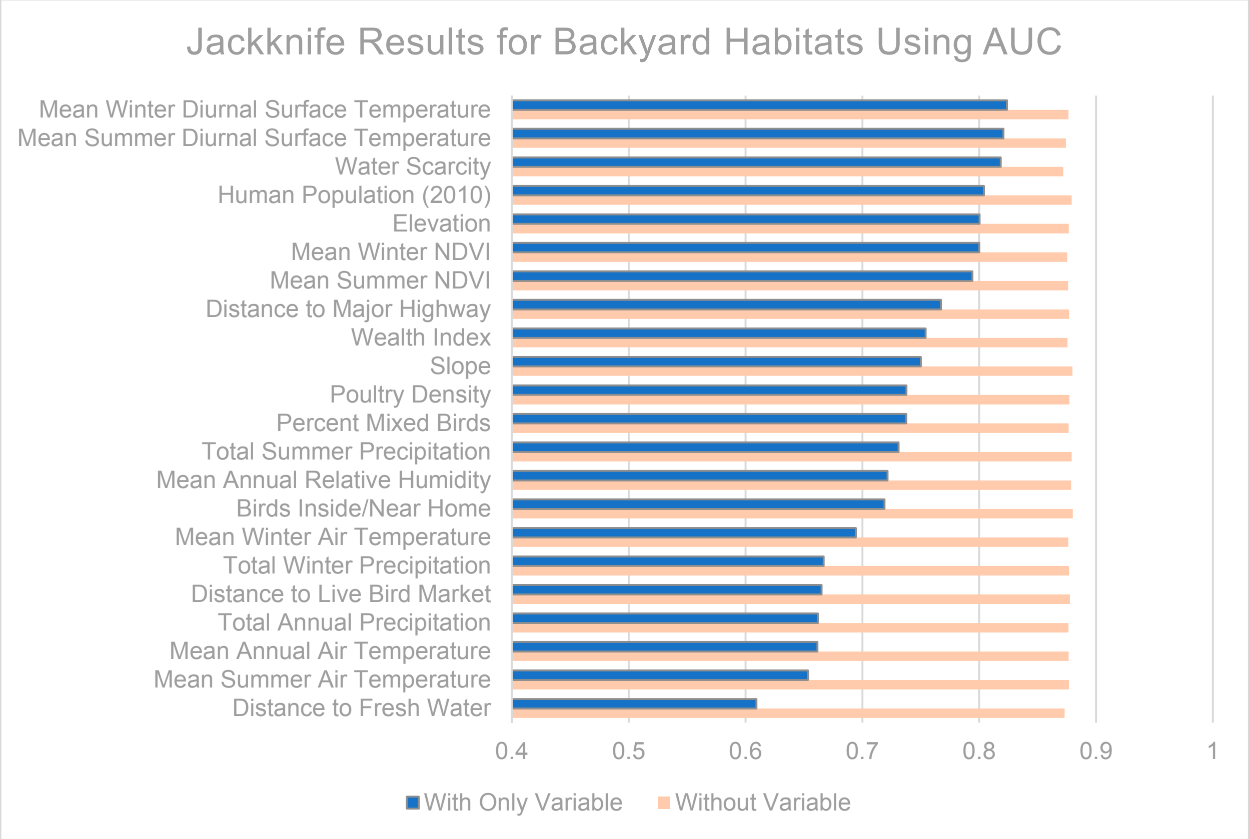

Figure S4. Jackknife results for backyard habitats.

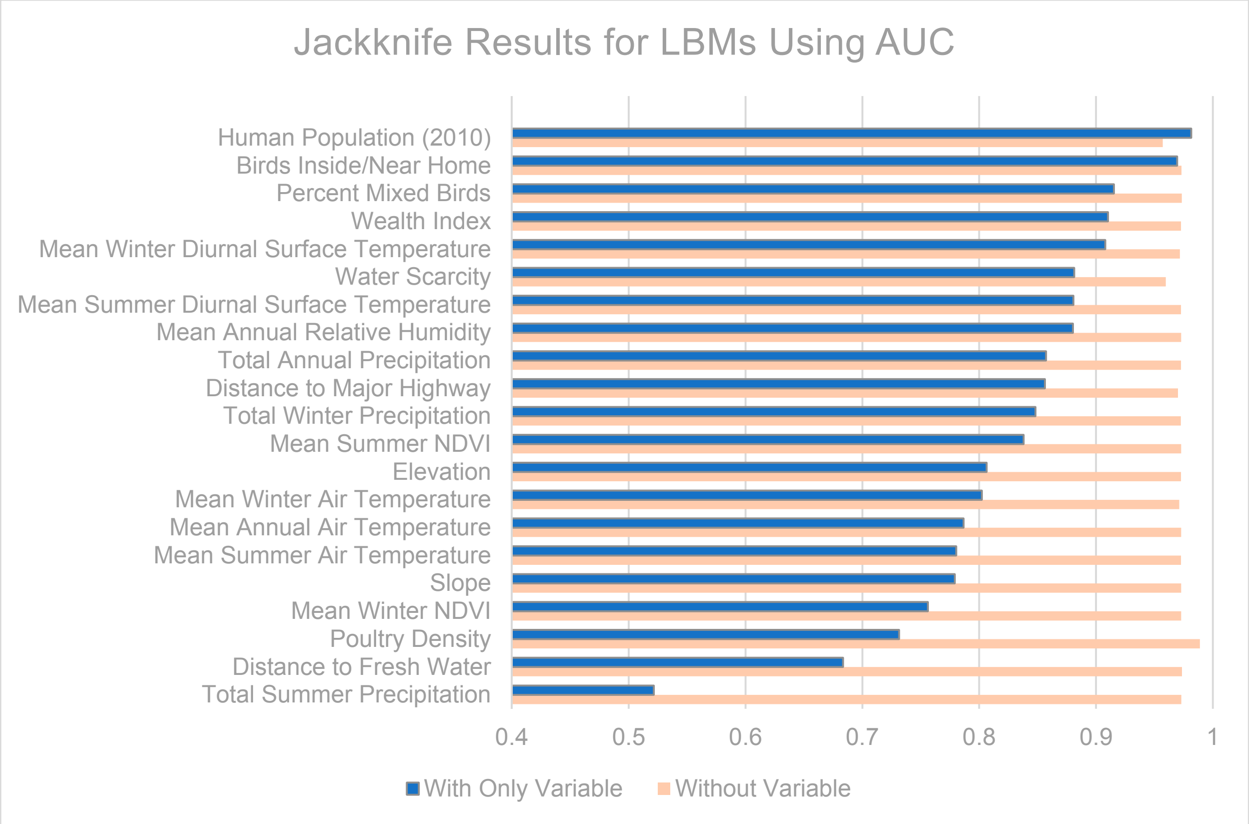

Figure S5. Jackknife results for LBM habitats.

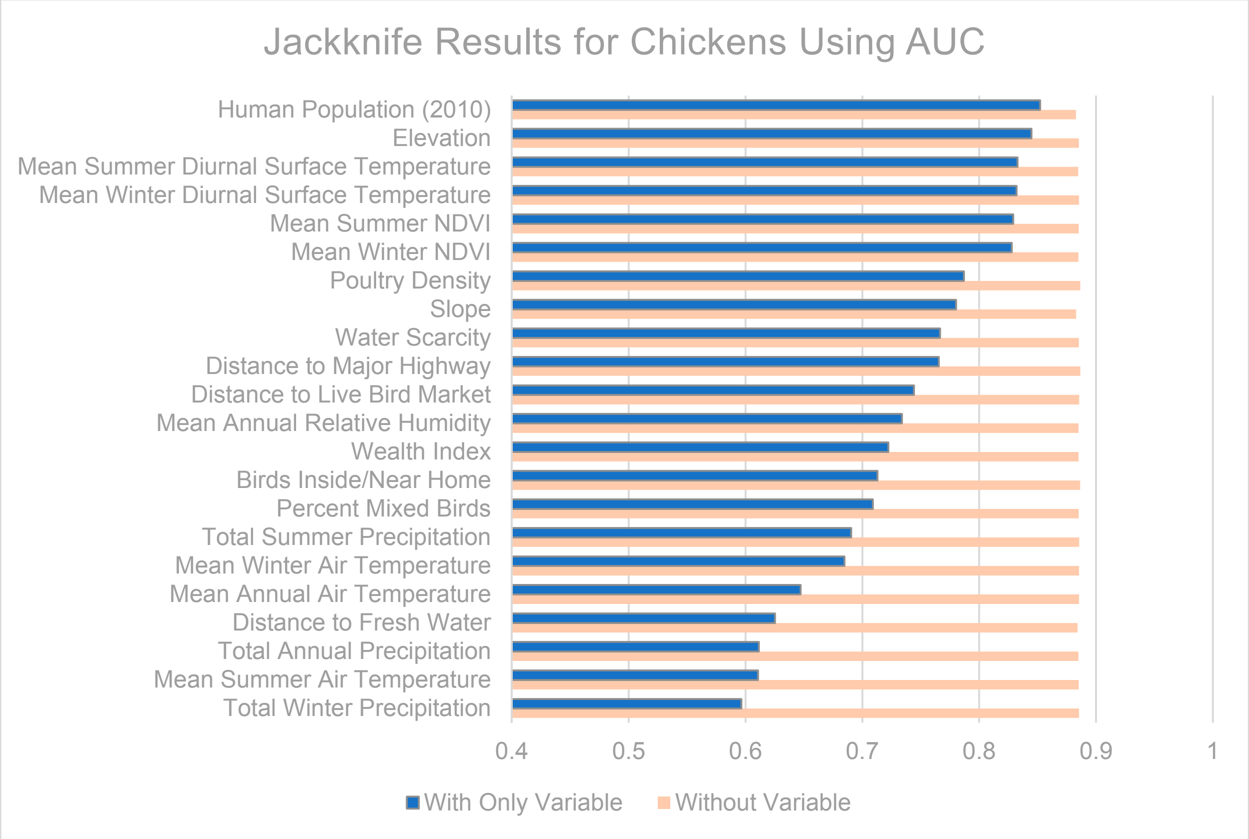

Figure S6. Jackknife results for chicken hosts.

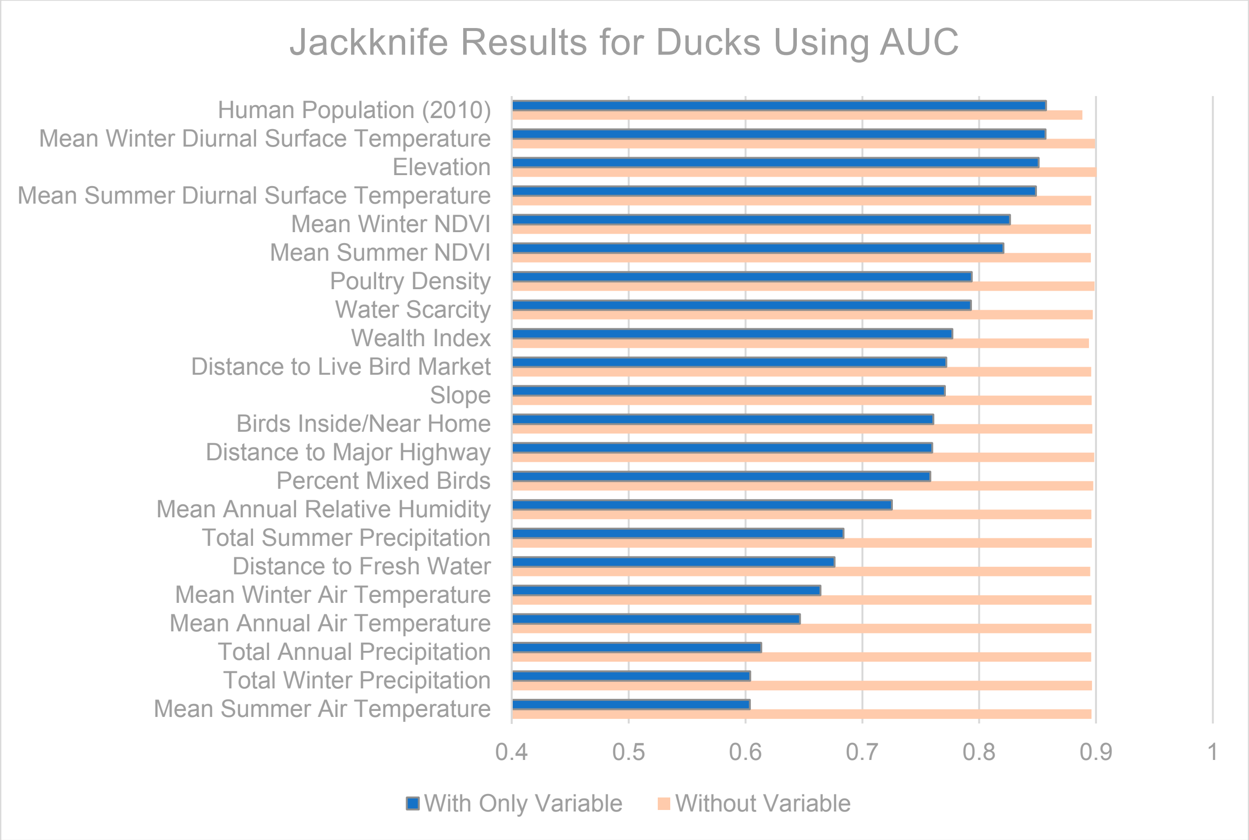

Figure S7. Jackknife results for duck hosts.

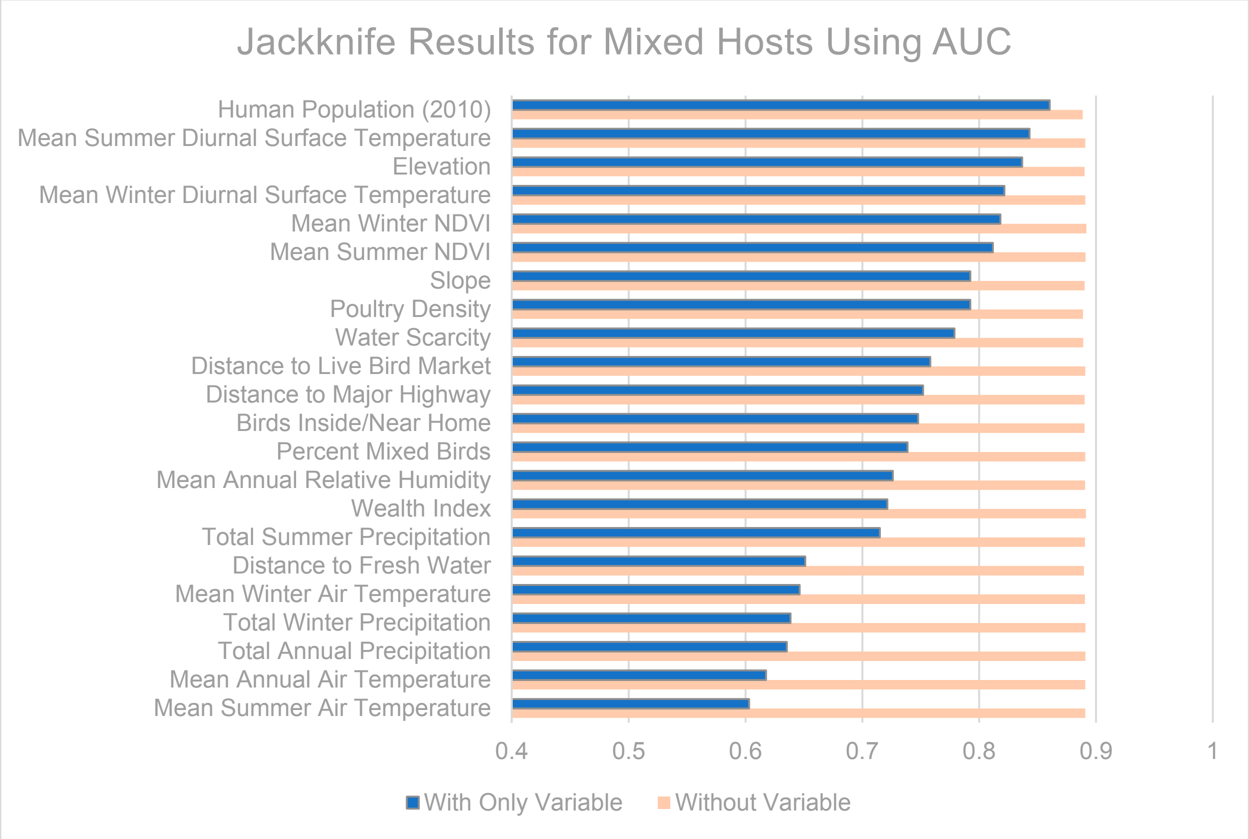

Figure S8. Jackknife results for mixed (chicken and duck) hosts.

The AUC results for all of the models are reported in Table S2. All models tested achieved AUC scores above 0.5, with most above 0.9, although small sample sizes were again a cause for concern, and validation with independent co-infection data was not possible.

Table S2. Area under the ROC curve (AUC) for avian influenza niche models

Dataset (Subset)	AUC
H5N1 (Backyard)	0.886
H5N1 (Commercial)	0.930 ‡
H5N1 (LBM)	0.974 ‡‡
H5N1 (Chickens)	0.905
H5N1 (Ducks)	0.908
H5N1 (Mixed)	0.911

Training Sample Size, ‡: <100, ‡‡: <30.

© 2016 by the authors; licensee MDPI, Basel, Switzerland. This article is an open access article distributed under the terms and conditions of the Creative Commons by Attribution (CC-BY) license (http://creativecommons.org/licenses/by/4.0/).
